# This time with feeling: recommendations for full-bodied reporting of research on dance

**DOI:** 10.3389/fcogn.2024.1385087

**Published:** 2024-07-18

**Authors:** Rebecca Elizabeth Barnstaple, Jessie Laurita-Spanglet, Jason Fanning, Christina Soriano, Christina E. Hugenschmidt

**Affiliations:** ^1^Department of Dance, York University, Toronto, ON, Canada; ^2^International Institute for Critical Studies in Improvisation, University of Guelph, Guelph, ON, Canada; ^3^Department of Theatre, University of Southern Maine, Gorham, ME, United States; ^4^Health and Exercise Sciences, Wake Forest University, Winston-Salem, NC, United States; ^5^Department of Theatre and Dance, Wake Forest University, Winston-Salem, NC, United States; ^6^Wake Forest Baptist Medical Center, Winston-Salem, NC, United States

**Keywords:** embodiment, interdisciplinarity, dance, aging, arts-based, arts and health, research design

## Abstract

Collaborations that employ methods from arts and sciences to address research questions through multimodal study design are becoming more frequent, as it is increasingly apparent that complex challenges require transdisciplinary solutions. These different modalities rely on interdisciplinary exchange while involving unique expertise in associated delivery practices. In human subject driven scientific research specifically, guidelines for arts-based interventions deserve detailed reporting to allow for fidelity, replicability, and uptake of innovation and results. Details such as frequency, duration, delivery method, expected outcomes, historical precedence, and instructor training are crucial, along with nuanced descriptions pertaining to embodied aspects of specific dance or movement style(s) and adaptations made for the population or study design. This Perspective Paper outlines the current state and challenges of reporting on dance interventions and makes recommendations based on our experience as teaching artists who work in research settings alongside researchers who collaborate with dance professionals.

## Introduction

Artistic practices are an integral aspect of the health and wellbeing of communities. Human cultures throughout history have established means of moving and being together that express crucial understandings of how to live in their unique times, spaces, and circumstances (Ness, 1992). Diverse dance forms are as nuanced, complex, and multifaceted as the societies in which they develop, and the embodied knowledge they contain speaks to how various cultures construct and nurture the individuals and relationships comprising their social fabric.

Increased incidences of health and anxiety disorders globally (COVID-19 Mental Disorders Collaborators, 2021) may be linked to a deterioration of embodied practices such as group and community dances that have long contributed to the formation and maintenance of human bonds (Tarr and Dunbar, 2023). Lack of social connection has been shown to pose a significant risk for individual health and longevity, as reported in the recent U.S. Surgeon General's Advisory [Office of the Surgeon General (OSG), 2023]. Loss of community spaces and fracturing of cultural and artistic practices has direct impacts on health and wellbeing contributing to mortality (Holt-Lunstadt et al., 2015), as these play a vital role in addressing challenges associated with loneliness and social isolation by fostering creativity, engagement, and relationships.

A recent report from the World Health Organization (WHO) on the role of the arts in improving health and well-being (Fancourt and Finn, 2019) calls for policy changes introducing or strengthening lines of referral from health and social care to arts programmes through pathways such as social prescribing, which connects people to non-clinical programs and services (Poulos et al., 2019). This report, which identifies a major role for arts in the prevention and promotion of health and the management and treatment of illness across the lifespan, makes over fifty references to dance programs and their associated benefits. This reflects the growing body of published research on dance interventions, which while promising, lacks specificity and depth in reporting parameters that apply to the embodied experience of dancing.

Publications specific to the benefits of dance and cultural movement-based programs for older adults (Sheppard and Broughton, 2020), particularly in relation to conditions such as Parkinson's (PD) and Alzheimer's Diseases (AD), have increased exponentially over the last 20 years. However, the heterogeneity of practices and outcomes makes meaningful comparison and definitive recommendation impracticable (Rice et al., 2024). A lack of clarity concerning specific aspects and attributes of interventions impedes replication and contributes to confusion in both scientific and lay audiences. For example, “dance therapy” has been used in research publications to refer both to artistic dance or “Dance for Health” (DfH) programs designed to have therapeutic effect, and Dance/Movement Therapy (D/MT), a creative arts modality. While both approaches are likely to have benefits, their precise mechanisms, methods, and facilitation style could differ significantly. DfH programs are defined by the International Association of Dance Medicine Science (IAMDS) as “holistic, evidence-based activities for the individual to manage and adapt to physical, mental, and social health challenges” whereas D/MT is defined by the American Dance Therapy Association (ADTA) as “the psychotherapeutic use of movement as a process which furthers the emotional, cognitive, physical and social integration of the individual”. DfH sessions are provided by “trained teaching artists” (IADMS), while D/MT requires graduate-level training and is a regulated profession in many states and countries. Confusion between these approaches makes it difficult to assess their full impact, limiting the development of targeted applications.

Research on dance interventions should (ideally) involve collaboration between dancers and dance educators, researchers, clinicians, and input from and consideration for participants. However, these stakeholders are generally accustomed to different manners of communication, primary objectives, and presentation formats, leading to crucial elements either being lost in translation or underreported. Our recent scoping review of the literature on dance interventions for older adults (Rice et al., 2024) outlines the heterogeneity of methods, populations targeted, intervention characteristics, and outcomes in dance research. In this companion piece, we outline the current state and challenges of reporting on the specifics of dance interventions and make recommendations for the dissemination of impactful science, drawing on our experience in developing and delivering dance-based protocols in research settings.

In the growing body of literature involving dance and health or aging, details related to the content and structure of the dance experience itself are sparse, when provided at all. This may be in part due to reporting conventions, but as teaching dance artists and research collaborators, we feel it is critical to acknowledge and include aspects of dance expertise and embodied knowledge within research publications. These details aid scientists in understanding the complexity that attends various dance forms, and better equip dance interventionists to translate their work to a broader community, ensuring replicability and appropriate application. The combination of physical, affective, cognitive, and social aspects of dance has resulted in a body of literature that is highly heterogeneous, with a broad range of outcome measures. Our review (Rice et al., 2024) identified over 2,000 papers and includes results from 114 studies (129 papers in total). While the bulk of these report improvements across motor and cognitive domains, the diversity in outcomes, study designs, and (lack of) details related to dance, presents substantial challenges to determining mechanisms involved in specific areas of improvement, impeding progress toward high-impact research.

Omissions in the characteristics of the dance interventions themselves are particularly glaring, as these are generally limited to reporting frequency and “style”, a term which masks a wealth of details related to the how the dance form is taught, learned, modified and experienced. Our review identified 41 different dance types/styles in the included studies, and many of these collapsed several forms under a broad category (for instance, “ballroom dance” included 5–11 different forms under one type). If each form were counted individually, there would be 68 forms represented across all studies, including diverse cultural forms from around the world, and nine forms specifically adapted for older adults or those with neurodegenerative diseases. Five studies compared two dance forms, and these included group, partnered and non-partnered dance (Rice et al., 2024). This reflects the “living” nature of dance, which is always changing.

A 2011 paper (Robb et al., 2011) outlined recommendations for reporting on music-based interventions, with the goal of improving transparency and specificity in that field. These recommendations were intended to support TREND and CONSORT guidelines while accounting for the variety, complexity, and uniqueness that are vital to the efficacy of arts-based interventions. The resulting checklist included seven different components: intervention theory, intervention content, intervention delivery schedule, interventionist, treatment fidelity, setting, and unit of delivery. These elements were chosen as those that were (1) relevant across a wide range of music-based interventions; (2) essential for interpretation of outcomes; and (3) necessary for replication and translation. Taking this music-based framework as our inspiration, we offer the following guidelines for reporting on characteristics of dance-based interventions, adapted to reflect the physical practice of dancing: choice of intervention/style, interventionist, setting and equipment (mode of delivery), structure/framework of dance intervention, movement details, music, and fidelity. A checklist (Table 1) is offered in the spirit of Robb, Burns and Carpenter to support adoption by researchers.

**Table 1:** Checklist for reporting for dance-based interventions.

**Dance based intervention reporting criteria**
Domain 1: Rationale for choice of dance intervention
a. Existing evidence for influence of the intervention on outcomes in relevant populations b. Clear hypothesis for how the intervention affects specific outcomes in the population
Domain 2: Interventionist
a. Experience with the intervention (training, background, qualifications) b. Experience with the participant population c. Role in the intervention (development? teaching? facilitating?)
Domain 3: Setting
a. Space is accessible and suited to the intervention b. Spatial and material modifications were made as and if needed
Domain 4: Structure/Framework of dance intervention
a. Class (or other) format? b. Group? Partnered? c. Session flow (warmup, cooldown, etc) d. Duration e. Frequency/number of sessions f. Relationship to previous/next class (learning new material vs. rehearsing)
Domain 5: Movement
a. Dance form b. Objective/focus (Theme/Goal) c. Agency d. Body/Space e. Timing/tempo f. Level of exertion g. Improvisation vs. Choreography h. Modifications/adaptations employed to suit study population i. Scaffolding/skill building/continuous challenge j. Safety
Domain 6: Music
a. Genre/origin b. Who chose the music and why? c. Live or pre-recorded d. Volume e. Counted? f. Relationship to movement g. Same from class to class?
Domain 7: Fidelity
a. Interventionist training b. Monitoring/record-keeping/checklists c. Participant feedback d. Ecological validity—methods

## Recommendations for dance-based intervention reporting

### Domain 1: rationale for choice of dance intervention

There should be clear reasoning provided for specifics of the dance intervention, including (a) references to previous research using this dance form along with an adequate treatment of historical and cultural associations, and (b) a hypothesis for how elements of the form may impact specific target outcomes for the study population. In cases where there is limited or no previous research on the use of a particular form, it may be appropriate to provide justification from adjacent literature demonstrating reasons for the choice, such as examples from music or physical therapy research; details related to the condition or disease state being studied; or known responses to other creative arts and/or health interventions.

Attention to the cultural background of researchers, interventionists and participants in relation to the dance form(s) utilized and reporting on acceptability, familiarity, and meaning associated with the dance is of high importance. Many dance forms may (and do) contain embodied knowledge of cultures, times, and places, and to ignore this aspect risks leaving out crucial information. The manner of cultural transmission (teaching style) comes into play here as well—is the dance form taught or shared in a circle or are participants arranged in front-facing lines? Does a teacher model a movement that is repeated, or is there group exploration and creativity? How are key concepts demonstrated, displayed, of imbued? Does the style primarily rely on visual, auditory, or kinesthetic feedback during the learning process? Dances evolve in specific historic contexts and their manner of teaching and execution reflects these origins. Knowledge of and respect for cultural aspects of dance is key to providing adequate descriptors.

**Figure 1:**
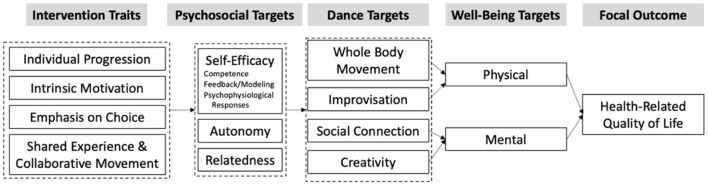
Intervention Mapping Example - Intervention mapping links specific traits of the intervention to both psychosocial and behavioral (dance) targets. These in turn are linked with other outcomes of interest, such as physical and mental well-being or health-related quality of life. Intervention mapping is one way to represent rationales related to the choice of (specific) dance forms in interventions.

There should be a clearly stated hypothesis as to the relationship between outcomes of interests and the putative mechanisms involved, including specific characteristics of the dance form and manner of delivery (Figure 1). This explication of “active ingredients” in the intervention can help disambiguate effects related to various intervention traits such as those outlined above; it can also contribute to less ambiguity in protocol design and assist with the adaptation of dance forms to research settings. How do traits in the intervention design relate to target outcomes through engagement with various aspects of dance? This allows for greater specification of discrete mechanisms and measurables, and most importantly, demonstrates the central importance of dance characteristics in modifying outcomes.

An example of this type of detail from a study included in our recent review (Rice et al., 2024) outlines the rational for using Greek traditional dance (Sofianidis et al., 2009) in relation to the study's hypothesis and target population. The authors justified the use of this form in relation to their outcomes of interest (static and dynamic postural sway) as it involves self-imposed perturbations of the postural control system. The dance form was culturally relevant for study participants (Greek older adults), and psychosocial benefits for the specific population had been previously demonstrated. However, despite providing details related to the hypothesis and dance style, and extensive information on measurement devices and results, this study provides almost no information at all on how the dance experience was led, taught, or facilitated, making replication of findings near impossible. This leads to the next domain, which in our experience is critical, while least frequently addressed in research papers—the background and approach of the person or people leading the intervention.

### Domain 2: interventionist

Research from education has clearly demonstrated that the effectiveness of both teacher and teaching style dramatically impact student learning and outcomes. Burroughs et al. (2019) identified several measures associated with higher student achievement: the teacher's experience, their professional knowledge, and the provision of opportunities to learn. These measures equally apply to teaching dance, which requires specialized skills and methods acquired through years of experience and training. A highly effective teacher or teaching model could dramatically alter outcomes; thus, the interventionist(s) role and potential influence cannot be understated in relation to the embodied aspects of dance-based interventions.

Adequate detail should be provided as to (a) previous experience with the dance or movement form used in the intervention (what is their training, background, qualifications); (b) experience with and knowledge of the participant population; and (c) clear specifications of the duties and role played in the design and delivery of the intervention itself—were interventionists involved in the development or design or the method used in the study? Who decided on the teaching/facilitation approach or pedagogical style? Is sufficient and appropriate time provided for instruction, demonstration, and feedback to participants? Descriptions of the interventionist's experience and approach should include a level of detail that supports replication. For instance, if the intervention was led by a trained teaching artist, were they teaching in a familiar style or form or was the approach used in the study new to them? In cases where the intervention is delivered by an allied health professional (such as a physiotherapist or occupational therapist), their level of experience and comfort with teaching dance, or any additional training provided to support delivery of the intervention should be reported. A study that attempts to recreate conditions previously documented may be flawed if the intervention is led by a less experienced interventionist, or one with a very different approach to teaching dance.

In terms of professional experience, knowledge is required of the participant population, to facilitate and promote safety while providing a meaningful experience with embedded adaptations as needed. If a range of adaptations are allowed for and provided in relation to various levels of mobility or experience, these should also be reported in terms of scope, frequency, and manner of presentation (for instance, if there are multiple teachers/models providing variations on a movement idea or exercise). If the interventionist(s) delivering the protocol also developed or contributed to it, they should have the opportunity to provide an outline of the class or experience structure and pedagogical approach. This should be sufficiently detailed to allow for the experience to be replicated by a different teaching team at another site. If the protocol was not developed by the intervention team, there should be an opportunity for the feedback and input of dance professionals to ensure that aspects of dance experience are suitable, adequate, appropriate, and acceptable. The provision of links to manuals or video examples, or the use of codified or culturally established forms can help clarify teaching style.

### Domain 3: setting

Location of the intervention or class can affect the participant experience. If the environment is not a pleasant or easily accessible one, it may impact adherence and fidelity of participants along with the morale of the intervention staff and volunteers. A dedicated dance studio is not the same as a room in a busy community center, or a quickly repurposed clinical area. Suitable workspace is as important in dance as it is for other disciplines. Considerations can be divided into two aspects that should be reported: a description of the space itself and any challenges or limitations, and any modifications of the space made by the interventionist to enhance safety, security, or improve the experience of participants. Examples of the first type of consideration include access to parking, bathrooms, water, and the space itself, especially if there are any mobility challenges for participants. The second type of consideration includes any modifications made to promote or inhibit participation, such as ensuring privacy and/or responses to limitations impacting critical elements such as music volume or flooring.

### Domain 4: structure/framework of dance intervention

This is the domain most frequently reported with some degree of detail in the literature; however, there is room for improvement as each of the elements have a wide range of nuance. The results of our review (Rice et al., 2024) found that dance studies ranged in frequency from less than one class per week to five classes a week; class length ranged from 30 to 120 min; and the duration of interventions ranged from 2 weeks to 18 months. There is also a wide range of variability possible regarding the intensity of the dance intervention, as a whole and in part. This is crucial information, as research in the field of exercise science indicates that intensity has direct bearing on the efficacy of the intervention in relation to various outcome measures, particularly those associated with physical and cardiorespiratory fitness (Garber et al., 2011). Diverse dance traditions, however, involve different structures of learning and execution. For example, some classes may start with a short warm up and then go into intense full-bodied dancing, while others may move more slowly and progressively. Some classes include a cool down while others end with learning a longer movement phrase. The flow and structure of sessions should be described in as much detail as possible to allow for replication—if there is a warm-up, what is involved and how is it conducted? It is also important to note the relationship between sessions—is there progression, repetition, or exploration? Movement transmission is not a homogenous process across cultural forms; dances can be circular or linear, improvised or choregraphed, created by the group or individuals or modeled by an expert. Intervention teams should report on the overall structure of learning and performing the dance(s) as well as relational elements that may be involved such as partnering, touch, and weight sharing. Interactional elements, if present, require additional information such as whether partners are self-selected or randomly assigned, familiarity between partners both within the class context and outside of it (dancing with a life- or care partner is a different experience than learning how to move with a relative stranger), and if there are various levels of experience in the class and/or interactions between participants with varying degrees of skill. This extends to the role of volunteers or support persons who interact with participants during the session. Are there different visual experiences entailed in the structure—do participants see and respond to each other, or only the instructor? Elements such as eye contact, touch, and auditory feedback from the teacher, other participants, or physical elements (such as tap shoes or floor percussion) could profoundly influence the experience of the dance and the outcomes of associated research.

### Domain 5: movement

The types of movements practiced within various dance forms are of primary importance and are surprisingly underreported. In our scoping review (Rice et al., 2024), the range, type and quality of movement were scarcely mentioned, as it is perhaps assumed that these are standard to various forms. While we acknowledge that detailed descriptions can be difficult given the variability and sheer quantity of movements encompassed within a dance experience, movement vocabulary is of great importance for replicability purposes. Information should be provided as to the most salient aspects of movement involved in the specific practice, with enough detail that the dance experience could be meaningfully reproduced in another setting.

Characteristics of movement have been thoroughly inventoried in movement analysis and notation systems used in dance, such as Laban Movement Analysis (LMA), which includes ways of describing and documenting use of Body, Effort, Space, and Shape (Cruz-Garza et al., 2014; Tsachor and Shafir, 2019). Study designs should provide detailed descriptions of potential and recorded movement repertoires along with how movement is described and/or transcribed in the research protocol. Examples of movement descriptions could include full body movement vs. detailed hand or arm gestures, planes of movement (sagittal, horizontal, vertical), levels and level changes (floor, seated standing), various forms of locomotion (jumping, rolling, turning), temporal dynamics and range, spatial orientation, and other details related to expressivity: how the movement is performed.

Related details pertain to whether or not there is an objective focus, theme or goal to sessions or specific movements; how engagement with the movement structure interacts with self-efficacy (Waugh et al., 2023); the use of choreographed material vs. improvisational prompts; skill acquisition that is required or facilitated by the method; any modifications that are made for various motor or other abilities in the class (was everyone doing the same thing?); how were challenges faced; was there a continual sense of challenge; did the instructor/class cultivate a sense of success/safety for participants; were a range of movement choices provided or allowed.

### Domain 6: music

Dance traditions and forms have different relationships with music, ranging from integral to informal, oppositional to non-existent. In the most common case, music is inextricably linked to the dance form and the two must be experienced and practiced together—in many languages, music and dance are connoted by a single term (Fitch, 2016). Dance styles or phases of learning that do not include music are less common and for this reason should take additional measures to describe how movement unfolds in time or creates its own rhythmic structure. Some dance forms may not use music at all, or at least not metered music, such as specific folk dances (Nijemo Kolo), dance theater (Butoh), and contemporary dance (Contact Improvisation). Many forms of dance shift and change the relationship with music depending on the needs of participants or setting, and silence can be used in addition to or alongside music, particularly for learning, warm-up, or cool-down phases. In the absence of, or in preparation for musical accompaniment, many dances are “counted” (5, 6, 7, 8) as a teaching strategy; as counting provides a provisional rhythmic structure, it may elicit entrainment that can impact learning and should be reported. Additional details include whether the music is live or pre-recorded, genre and selection process, volume and tempo (steady or changing), the relationship of movement to music, and if there are periods of class/session that involve teaching without music and their duration. Reports should also include whether the music and the manner it is used remains the same throughout each class and over the progression of the intervention.

### Domain 7: fidelity

Interventionists (especially teaching artists who are new to working in research settings) may require specific training in elements such as privacy, fidelity, and reporting to ensure proper execution of protocols. What monitoring is carried out to ensure treatment fidelity, to what extent is the movement protocol “fixed” or pre-determined, and what processes are in place to report deviations or adaptations? Additionally, there may be value in including teaching artists in study design and development, considerations around outcome measures, and the ethical review process, as they may have relevant concerns or suggestions that could be usefully incorporated. Training for teaching artists on specific concerns relevant to working with older populations can be crucial, as concepts such as empathy, expectations around success, the importance of not infantilizing adults, and treating research participants as dancers rather than patients can be critical for success. Having a process for receiving and responding to participant feedback helps to ensure that these criteria have been met and allows for adjustments when necessary.

Experienced teaching artists have significant insight as to how aspects related to movement and group cohesion are influenced by their work. While dance professionals and scientists may use different language to describe effects and interactions, teaching artists often have nuanced perspectives related to impacts of the intervention and how different components of the dance form or pedagogical choices influence the outcomes they see, which scientists are trying to measure. The involvement of artists as an integral part of the research team can lead to study designs that are well informed in terms of intervention detail, outcome measures, and analysis. Considering the lead artist or interventionist as an equal investigator validates their embodied expertise. This creates space for the intervention to be thoughtfully planned and executed in relation to the scientific priorities within the parameters of the study design.

## Discussion

We have outlined a detailed checklist of recommendations for reporting on dance-based interventions that we hope will be useful to researchers, practitioners, and knowledge users by supporting greater transparency and transfer of methods and results in this emerging field. Our goal is to highlight the complexity and diversity of practices associated with dance that are often missing from reports in research literature—while it may not be practical to fully address every element of the checklist in all circumstances, we hope to encourage further engagement with the embodied elements of dance in research design and reporting.

We also recognize that the fundamentally embodied nature of dance does not always lend itself to lexical description. While attention to the elements outlined in the checklist and intervention mapping model above would undoubtedly contribute to deepening understandings of the nuanced and diverse practices involved in dance research and their effects, there is a need to adopt other models of research dissemination suited to capturing and portraying the finer details of dance experiences. For this to happen, we need to move beyond print journal articles as the foremost and only format acceptable for rigorous sharing of study results. As we hope has been made clear, current conventions around reporting are suited to sharing some details but poor in representing others. Reading about a dance, whether in the context of research or culture, provides little in the way of embodied detail about the first-person experience of dancing, or what it was like to *be* there. Multiplatform journals that include video such as the Journal of Embodied Research are a positive sign of evolution in this direction, as is the Journal of Visual Experiments (JoVE) which provides space for scientists to publish experimental methods in video format. A next step, which is beginning to be utilized by some authors, is the inclusion of weblinks for multimedia or video elements in “mainstream” journals as a complement to text-based elements, much as Tables and Figures serve to expand or explain elements currently.

Until such time as new mediums for dissemination become common practice, there are aspects of these recommendations that may be undertaken by various actors to improve reporting immediately. For investigators, greater attention to the suggested elements would improve the quality of reporting and facilitate adoption of protocols and better understanding of results. Collaborations between researchers with different backgrounds in the sciences or dance can be strengthened by acknowledgment and respect for equal but different forms of expertise—there is much to be learned from sharing across these fields, both of which are committed in their own ways to investigation and analysis. For journals and other forms of publication, a more rigorous attention to the level of detail provided on embodied aspects of interventions, and an openness to new forms of representation for experiential elements would facilitate uptake and understanding of dance-based interventions within and beyond the field. Engaging dance and movement professionals in the peer-review process could help to ensure that these elements are adequately described and replicable.

There will always be challenges in reporting *post-hoc* on embodied experiences, as much of the detail involved remains elusive and ineffable. Due to the diversity, complexity, and nuance involved in all instances of dancing, there will inevitably be details that elude description in any format. Capturing elements such as mood, sense of accomplishment, and other “soft” aspects can be crucial to understanding what mattered in the moment; what's more, this can vary between participants and shift rapidly even in the same person. Humanities-based methods such as phenomenological ethnography are suited to identifying these elements, and if used in combination with scientific measures and models, may lead us closer to understanding the most impactful aspects of embodied experiences. Evolving experimental designs that include both biological and affective indicators such as hormone changes, epidermal conductivity, and heart rate along with mobile neuroimaging, phenomenological interviewing, and “thick” description expanded by detailed real-time media such as video and motion capture are indicating future directions for research that has the complexity and power to grapple with bodies in motion, engaged in meaningful, creative expression.

## Data availability statement

The original contributions presented in the study are included in the article/supplementary material, further inquiries can be directed to the corresponding authors.

## Author contributions

RB: Investigation, Methodology, Conceptualization, Writing – original draft, Writing – review & editing. JL-S: Conceptualization, Investigation, Methodology, Writing – original draft, Writing – review & editing. JF: Conceptualization, Visualization, Writing – review & editing. CS: Conceptualization, Funding acquisition, Investigation, Methodology, Supervision, Writing – original draft, Writing – review & editing. CH: Conceptualization, Funding acquisition, Investigation, Supervision, Writing – review & editing.
